# Delayed Vegetation Mortality After Wildfire: Insights from a Mediterranean Ecosystem

**DOI:** 10.3390/plants14050730

**Published:** 2025-02-27

**Authors:** Giulia Calderisi, Ivo Rossetti, Donatella Cogoni, Giuseppe Fenu

**Affiliations:** 1 Department of Life and Environmental Sciences, University of Cagliari, 09123 Cagliari, Italy; giulia.calderisi@unica.it (G.C.); d.cogoni@unica.it (D.C.); 2 Research Centre of S. Teresa, ENEA (Italian National Agency for New Technologies, Energy and Sustainable Economic Development), 19032 Lerici, Italy; ivo.rossetti@enea.it

**Keywords:** vegetation recovery, Montiferru massif, Sardinia, megafire, Mediterranean Basin

## Abstract

Wildfires, one of the most important ecological disturbances, influence the composition and dynamics of ecosystems all around the world. Changes in fire regimes brought on by climate change are making their effects worse by increasing the frequency and size of fires. This study examined the issue of delayed mortality at the species and community levels, concentrating on Mediterranean forests dominated by *Quercus ilex* and *Quercus suber*. This research examined areas lacking spectral recovery following a megafire, which, although relatively small compared to the total burned area, represented significant ecological disturbances. The results highlighted distinct post-fire dynamics at both the woodland and species levels. *Q. ilex* experienced higher delayed mortality, particularly in areas of lower fire severity (NR), likely due to increased intra-specific competition. Because of its thick bark, which offers stronger fire resistance and encourages regeneration even in high-severity fire zones (HR), *Q. suber* showed greater resilience. Responses from the shrub layer varied, and some species, such as *Pteridium aquilinum* and *Cytisus villosus*, showed post-fire proliferation. To improve our knowledge of ecosystem resilience and guide forest management in fire-prone areas, these findings highlight the intricacy of post-fire ecological processes and the need to integrate species-specific features with more general community-level patterns.

## 1. Introduction

Wildfires are considered among the most important ecological disturbances that influence the structure and functions of ecosystems in many areas of the world (e.g., [1,2,3,4,5]). Changing fire regimes, driven by warmer temperatures and shifting precipitation patterns, have made large fires an almost annual occurrence in many regions of the world [5,6]. Wildfires impact ecosystems and landscapes by causing biomass and habitat loss and affecting carbon emissions, pervasive community changes, and land-use shifts [7]. However, natural fire also plays a crucial ecological role by shaping and enhancing forest structural complexity and biodiversity through varying severities, geomorphic factors, and vegetation types [5,8,9,10]. In fact, fire regulates species dominance and tree density in fire-adapted and fire-dependent ecosystems [11], resulting in a habitat that supports a variety of plant and animal species that would not be able to survive without fire [12].

It is widely known that in fire-prone areas, such as the Mediterranean Basin, the natural vegetation exhibits resilience to fire [13]. Many Mediterranean plant species have evolved strategies where they sacrifice their above-ground parts during a wildfire to ensure the survival of the species. Post-fire vegetation regeneration primarily relies on the emergence of new shoots from surviving underground plant components or on the germination of seeds to withstand high temperatures [14,15,16,17]. In general, post-fire vegetation regeneration is greatly dependent on fire severity (e.g., [18,19]). Fire severity is defined as wildfire-induced changes to the soil surface and vegetation conditions, and it is influenced by a variety of elements, such as terrain slope, pre-disturbance vegetation structure and composition, weather and climate conditions, and fuel characteristics, among others [20,21,22,23,24]. High-severity fires are characterized by complete canopy mortality and the burning of the entire top layer of soil, whereas low-severity fires result in the loss of ground and understory plants [23,24]. In addition, the recovery process after a wildfire can vary widely, depending on several factors, such as plant traits, environmental factors, vegetation type (e.g., [25,26,27,28]), climate and weather (e.g., [29,30,31,32]), topography (e.g., [25,31,32,33,34,35]), distance to unburned patches and seed sources [36,37,38], and soil properties [39].

In some cases, it may take years or even decades for the ecosystem to return to its pre-fire condition [40] or show a rapid recovery in a short time frame [17]. Tree species that withstand medium- and low-severity forest fires exhibit various adaptive responses, such as deep root systems and epicormic or basal sprouting [41,42,43,44]. These specific responses allow plants to avoid, resist, or tolerate fire (at the individual, community, or landscape level) [45]. For example, some plant species have developed thick bark or serotine cones that need fire to open and release seeds [46]. The cork oak (*Quercus suber* L.) has developed a thick cork bark that serves as a fire-resistant barrier, enabling epicormic resprouting even after high-intensity fires, an adaptive trait unique among European tree species [47,48]. Additionally, broadleaf species exhibit significant sprouting following fire events [49]. Moreover, the ecosystem type plays a critical role in post-fire vegetation regeneration [18]. By increasing tree mortality, wildfires can drastically lower above-ground biomass in humid tropical forests, especially the Amazon [50,51,52]. In some ecosystems, on the other hand, plants can rebound considerably more quickly; for instance, some coniferous trees evolved to withstand fire and employ the flames to disperse their seeds, as the heat causes the opening of cones [53]. Frequent seasonal fires contribute to the preservation of the biological richness, species composition, and structure in savannas [54]. Fires can even be necessary for canopy regeneration; a decline in the sequoia population was observed when fires were suppressed in California [55]. Fire-induced injuries can affect tree vigor, alter water-use efficiency, and result in either transient or lasting growth reductions, which may ultimately result in tree mortality [56].

Due to land abandonment and global warming, wildfire events have become more frequent and severe in the last few decades [57,58,59]. In the context of climate change, wildfire frequency and intensity are expected to increase, particularly in the Mediterranean Basin, which is known to be among the regions most impacted by future heat waves and droughts [15,60,61]. This finding raises concerns regarding the potential adverse consequences on post-fire vegetation recovery dynamics, biodiversity, traditional landscapes, ecosystem functions and services, and, consequently, human well-being [13,16]. In response, in recent years, extensive efforts have been dedicated to investigating different aspects of wildfires. However, while much research has focused on the overall impacts of wildfires, the dynamics at the edges of burned areas are often overlooked. Particularly at the edge of the burned region, intricate dynamics and challenges converge, making these areas essential to understanding the broader ecological effects of wildfires [62].

Determining the edge of a burned region is challenging due to technical limitations. In the case of vast fires, the remote sensing approach is essential for rapidly mapping the edges of burned areas and defining the heterogeneity of fire severity levels and post-fire vegetation recovery [62]. Complex fire boundaries create highly variable edges that can affect the structure and composition of the surrounding forest, extending deeper into the forest and resulting in a wider, more gradual transition zone [63,64]. The structure of these edges changes over time, creating dynamic boundaries that gradually impact the surrounding plant communities [65]. Furthermore, tree mortality and regeneration are key processes in shaping these edges [65]. Generally, fire severity close to the perimeters of burned areas is low to moderate [65,66,67]. As a result, the lower fire severity along the edges makes it more difficult to clearly determine the boundary of the burned area since the spectral discrepancy between burned and unburned vegetation becomes less obvious [68]. Additionally, fires with low or moderate severity often cause delayed mortality in damaged trees. This fire-triggered process may result in the loss of huge portions of phytomass, even for various years after the extinction of the fire. Since the severity of the fire is usually lower near the perimeters of a wildfire, delayed tree mortality often occurs around these areas [69].

In our previous study [17], we investigated the short-term recovery of Mediterranean vegetation following a megafire, demonstrating that the vegetation initiates regeneration within the first year, despite high fire severity levels. Recovery was mainly driven by basal and epicormic resprouting strategies, particularly in cork oak-dominated formations, as well as by seed germination induced by thermal shock [17]. In addition, this study highlighted anomalies in the marginal areas of the burned areas; over time, the discontinuity at the edge is expected to decrease as the contrast between nearby plant communities diminishes [70,71], leading to what is called ’edge softening’ [72]. Another important aspect is the unburned sides of fire edges, which are especially significant as refuges for flora and fauna and so contribute to their reestablishment in burned regions [65,73]. Given that the edges of burned areas are particularly prone to different ecological complications and dynamics, we subsequently examined post-fire vegetation recovery in these areas. We have demonstrated the presence of the delayed mortality phenomenon in these areas, and our results suggest that delayed tree mortality plays a significant role in low-severity fire zones close to the edges of the fire [62]. Furthermore, we observed that post-fire mortality also affected the shrub layer, which could be attributed to both fire-induced stress and additional environmental stressors [62]. Building on the results of our previous study [62], this new contribution focuses on exploring the delayed mortality phenomenon in detail, specifically analyzing this process at both plant species and community levels. In particular, the aim of the research is to determine whether different plant species and different forest types exhibit similar or distinct patterns of delayed mortality. By examining this issue in detail, this study aims to contribute to filling the knowledge gap regarding whether and how these patterns differ among plant species.

## 2. Results

### 2.1. Delayed Mortality at the Plant Community Level

**Figure 1:**
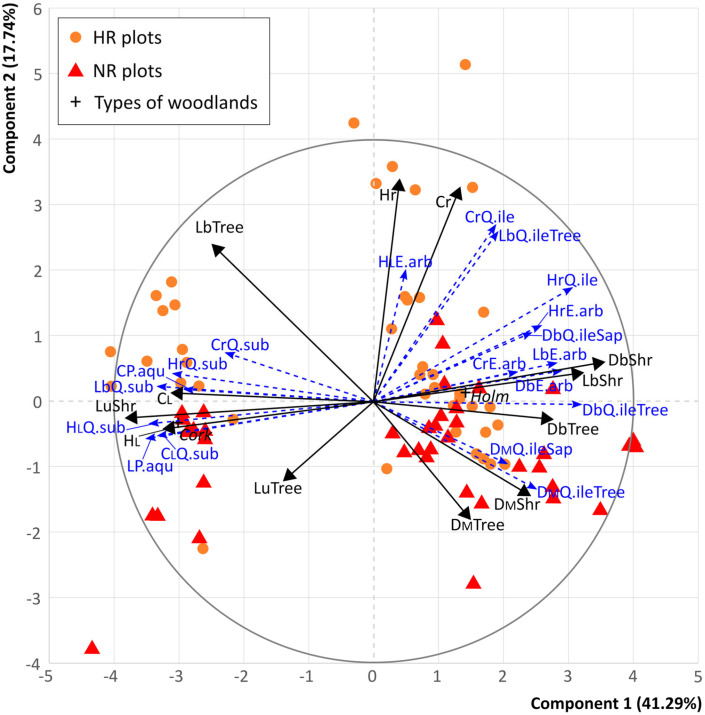
Biplot from the PCA showing the position of sampling plots (orange circles and red triangles), types of woodlands (black crosses) and superimposed active variables (plain black arrows), quantitative supplementary variables (dotted blue arrows), and the correlation circle. Only the supplementary variables with a minimum correlation of 0.5 with at least one dimension are shown in the biplot. CL = Cover of living plants, HL = Height of living plants, Cr = Cover of resprouts, Hr = Height of resprouts, Lb = Living burned plants, Lu = Living unburned plants, L = living plants (for *Pteridium aquilinum* only), Db = Dead burned plants, DM = Delayed mortality, Tree = Arboreal plants, Shr = Shrubs, Sap = Sapling, Q.ile = *Quercus ilex*, Q.sub = *Q. suber*, Q.ich = *Q. ichnusae*, E.arb = *Erica arborea*, P.aqu = *Pteridium aquilinum*, Cork = Cork oak woodlands, Holm = Holm oak woodlands.

PCA ordination revealed two main components, accounting for 41.29% and 17.74% of the total variance, respectively, which clearly highlighted two primary gradients (Figure 1). Sampling plots were clearly distributed along the first axes: cork oak woodlands on the left side of the graph and holm oak woodlands on the right. The second gradient, associated with the second component, was mainly related to the distribution of HR and NR plots.

Among the 12 active variables, distinct patterns emerged (Table S1 for details). Along the first component, cover and height of living plants, living burned and unburned trees, and living unburned shrubs were mainly associated with the cluster of cork oak woodlands, while living burned shrubs, dead burned trees, and shrubs were mainly associated with the cluster of holm oak woodlands. The second component was notably linked to the cover and height of basal resprouts, especially in the HR plots of holm oak woodlands. Delayed mortality of trees and shrubs was associated with first and second components and mainly with NR plots of holm oak woodlands, while living burned trees were associated with NR plots of cork oak woodlands. *Pteridium aquilinum* was primarily associated with cork oak woodlands. On the right side of the graph, the quantitative supplementary variables were mainly associated with *Q. ilex* and *E. arborea*. HR plots showed strong associations with the cover of resprouts and living burned *Q. ilex* trees, while the NR plots were mainly associated with delayed mortality of *Q. ilex*.

**Figure 2:**
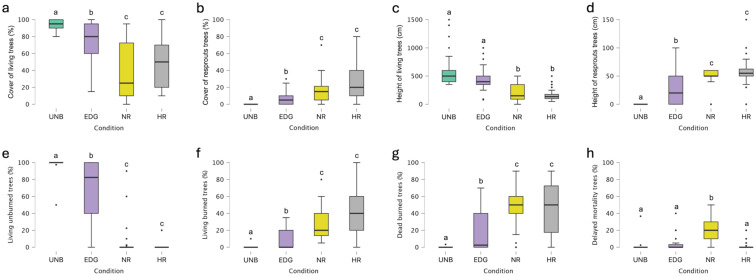
Boxplots of (**a**) cover of living trees, (**b**) cover of resprouted trees, (**c**) height of living trees, (**d**) height of resprouted trees, (**e**) living unburned trees, (**f**) living burned trees, (**g**) dead burned trees, and (**h**) delayed mortality trees, considering the total woodland level in unburned (UNB) plots, edge plots (EDG), no NBR recovery plots (NR), and high NBR recovery plots (HR). The boxes show the interquartile range, the horizontal bars show median values, and the vertical bars show the top and bottom 25% quartiles. Median values with different lowercase letters significantly differ at *p* < 0.05.

The Kruskal–Wallis test revealed statistically significant differences among conditions in terms of the cover of living trees at the woodland level (Figure 2a). Dunn’s post hoc comparisons revealed statistically significant differences (*p* < 0.05) among the various conditions, except for the comparison between NR and HR (see Table S2 for details). The NR plots had a lower average cover of living trees (39.20%), which was significantly less than that observed in the UNB plots (94.43%) (Figure 2a).

The Kruskal–Wallis test showed statistically significant differences among conditions regarding the percentage of living unburned trees at the woodland level (Figure 2e). Dunn’s post hoc comparisons indicated statistically significant differences (*p* < 0.05) among the different conditions, apart from the comparison between NR and HR plots (see Table S3 for details). The average percentages of living unburned trees in NR (4.47%) and HR (0.91%) were significantly lower than in UNB and EDG plots (98.81 and 68.83%, respectively) (Figure 2e).

Likewise, the percentage of living burned trees showed statistically significant differences among conditions, according to the Kruskal–Wallis test, at the woodland level (Figure 2f). Dunn’s post hoc comparisons exhibited statistically significant differences (*p* < 0.05) among the different conditions, except between NR and HR plots (see Table S3 for details). The average percentages of living burned trees in HR and NR (44.02 and 27.25%, respectively) were significantly different from those in EDG and UNB plots (7.77 and 0.23%, respectively) (Figure 2f).

The Kruskal–Wallis test revealed statistically significant differences among conditions for the percentage of dead burned trees at the woodland level (Figure 2g). Dunn’s post hoc comparisons showed statistically significant differences (*p* < 0.05) among the different conditions, except for the comparison between NR and HR (see Table S3 for details). The average percentages of dead burned trees in NR (46.55%) and HR (46.29%) were significantly higher than in EDG (17.16%) and UNB plots (0.08%; Figure 2g).

The Kruskal–Wallis test indicated statistically significant differences among conditions in terms of delayed mortality at the woodland level (Figure 2h). Dunn’s post hoc comparisons revealed statistically significant differences (*p* < 0.05) between NR and UNB plots, between NR and EDG plots, and between NR and HR plots (Figure 2h; see Table S3 for details). The average percentage of dead unburned trees in NR (21.72%) was significantly higher than in EDG (3.98%), HR (1.97%), and UNB (0.89%).

At the shrub level, the Kruskal–Wallis test showed statistically significant differences among conditions in terms of delayed mortality. The Dunn’s post hoc comparisons showed statistically significant differences (*p* < 0.05) between UNB and NR plots, UNB and HR plots, and EDG and HR plots (see Table S3 for details). The average percentage of delayed mortality in UNB (1.56%) was significantly higher than in NR (0.60%) and HR (0.30%), and the average percentage of delayed mortality in EDG (0.75%) was significantly higher than HR (0.30%).

### 2.2. Delayed Mortality at the Plant Species Level: Tree Species

**Figure 3:**
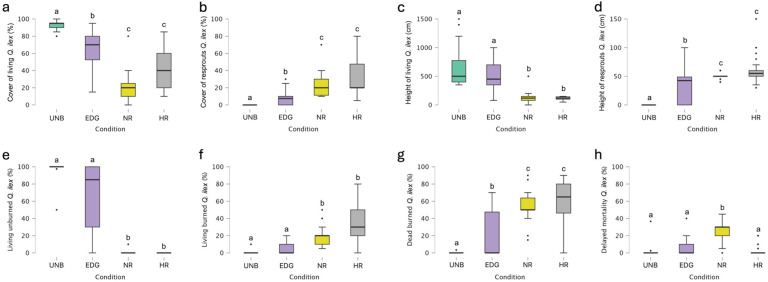
Boxplots of (**a**) cover of living *Q. ilex*, (**b**) cover of resprouted *Q. ilex*, (**c**) height of living *Q. ilex*, (**d**) height of resprouted *Q. ilex*, (**e**) living unburned *Q. ilex*, (**f**) living burned *Q. ilex*, (**g**) dead burned *Q. ilex*, and (**h**) delayed mortality of *Q. ilex* in unburned (UNB) plots, edge plots (EDG), no NBR recovery plots (NR), and high NBR recovery plots (HR). The boxes show the interquartile range, the horizontal bars show median values, and the vertical bars show the top and bottom 25% quartiles. Median values with different lowercase letters significantly differ at *p* < 0.05.

The most widespread forest species was *Quercus ilex*; the Kruskal–Wallis test revealed statistically significant differences among conditions in terms of the cover of living trees (Figure 3a). Dunn’s post hoc comparisons revealed statistically significant differences (*p* < 0.05) among the different conditions, except between NR and HR plots (see Table S2 for details). In particular, NR plots exhibited a low average cover of living trees (19.83%), which was significantly lower than in UNB plots (92.67%; Figure 3a).

The Kruskal–Wallis test also showed statistically significant differences among conditions in terms of the cover of resprouts (Figure 3b). Dunn’s post hoc comparisons showed statistically significant differences (*p* < 0.05) among the different conditions, apart from the NR–HR comparison (see Table S2 for details). EDG plots exhibited a low average cover of resprouts (7.83%), but it was significantly higher than in UNB plots, where resprouts were not present. The greatest average cover of resprouts was found in HR plots (32.33%), followed by NR plots (22.33%) (Figure 3b).

Likewise, the height of living plants showed statistically significant differences among conditions, according to the Kruskal–Wallis test (Figure 3c). Dunn’s post hoc comparisons revealed statistically significant differences (*p* < 0.05) among the different conditions, except for the comparisons UNB-EDG and NR-HR (see Table S2 for details). The average heights of living trees in NR (118.67 cm) and HR (113.67 cm) were significantly lower than in UNB (651.67 cm) and EDG plots (512.33 cm) (Figure 3c).

Regarding the height of resprouts, the Kruskal–Wallis test revealed statistically significant differences among the conditions (Figure 3d). Dunn’s post hoc comparisons showed statistically significant differences (*p* < 0.05) among the different conditions, except for the comparison between NR and HR plots (see Table S2 for details). The average height of resprouts in HR (61.17 cm) and NR (50.67 cm) was significantly different from those in EDG (27.67 cm) and UNB (where resprouts were absent) (Figure 3d).

The Kruskal–Wallis test revealed statistically significant differences among conditions for the percentage of living unburned trees (Figure 3e). Dunn’s post hoc comparisons showed statistically significant differences (*p* < 0.05) among the different conditions, except for the comparison between UNB and EDG and NR and HR (see Table S3 for details). The average percentages of living unburned trees in NR (0.33%) and HR (0.00%) were significantly lower than in UNB and EDG plots (98.25 and 63.50%, respectively) (Figure 3e).

Likewise, the percentage of living burned trees showed statistically significant differences among conditions, according to the Kruskal–Wallis test (Figure 3f). Dunn’s post hoc comparisons showed statistically significant differences (*p* < 0.05) among the different conditions, except for the comparison between UNB and EDG and NR and HR (see Table S3 for details). The average percentages of living burned trees in HR and NR (33.00 and 19.17%, respectively) were significantly different from those in EDG and UNB plots (5.17 and 0.33%, respectively) (Figure 3f).

The Kruskal–Wallis test revealed statistically significant differences among conditions for the percentage of dead burned trees (Figure 3g). Dunn’s post hoc comparisons revealed statistically significant differences (*p* < 0.05) among the different conditions, apart from the NR–HR comparison (see Table S3 for details). The average percentages of dead burned trees in HR (61.00%) and NR (55.50%) were statistically higher than those in EDG (22.50%) and UNB (0.11%) (Figure 3g).

The Kruskal–Wallis test indicated statistically significant differences among conditions in terms of delayed mortality (Figure 3h). Dunn’s post hoc comparisons showed statistically significant differences (*p* < 0.05) between NR and UNB plots, NR and EDG plots, and NR and HR plots (Figure 3h; see Table S3 for details). The average percentage of delayed mortality in NR (25.00%) was significantly higher than in EDG (5.50%), HR (2.67%), and UNB (1.31%) (Figure 3h).

**Figure 4:**
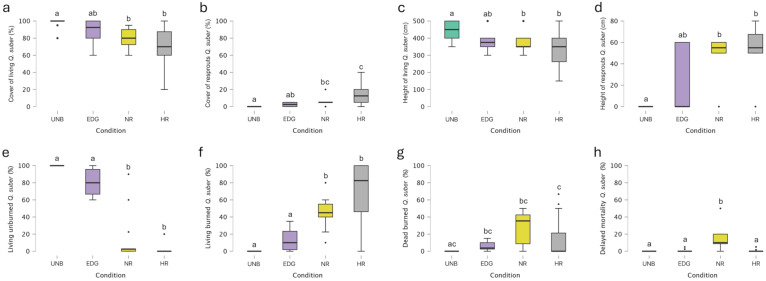
Boxplots of (**a**) cover of living *Q. suber*, (**b**) cover of resprouted *Q. suber*, (**c**) height of living *Q. suber*, (**d**) height of resprouted *Q. suber*, (**e**) living unburned *Q. suber*, (**f**) living burned *Q. suber*, (**g**) dead burned *Q. suber*, and (**h**) delayed mortality of *Q. suber* in unburned (UNB) plots, edge plots (EDG), no NBR recovery plots (NR), and high NBR recovery plots (HR). The boxes show the interquartile range, the horizontal bars show median values, and the vertical bars show the top and bottom 25% quartiles. Median values with different lowercase letters significantly differ at *p* < 0.05.

The second most important forest species of the genus *Quercus* that characterized the burned woods was *Quercus suber* L. The Kruskal–Wallis test revealed statistically significant differences among conditions in terms of the cover of living trees (Figure 4a). Dunn’s post hoc comparisons showed statistically significant differences (*p* < 0.05) between the average cover of living trees found in UNB (98.21%) and NR (80.71%), as well as between UNB and HR (68.57%) (Figure 4a; see Table S2 for details).

Considering the cover of resprouts, the Kruskal–Wallis test indicated statistically significant differences among the different conditions (Figure 4b). In particular, HR and NR plots showed the greatest average cover of resprouts (13.57 and 5.00%, respectively), and for Dunn’s post hoc comparisons, it was significantly higher than in UNB plots, where resprouts were absent (Figure 4b; see Table S2 for details).

Likewise, the height of living plants showed statistically significant differences among conditions, according to the Kruskal–Wallis test (Figure 4c). Dunn’s post hoc comparisons revealed statistically significant differences (*p* < 0.05) between the UNB (446.43 cm) and NR (371.49 cm) plots and between the UNB and HR plots (317.86 cm) (Figure 4c; see Table S2 for details).

In terms of the height of resprouts, the Kruskal–Wallis test revealed statistically significant differences across conditions (Figure 4d). The average height of resprouts in HR (52.14 cm) and NR (44.29 cm) was, for the Dunn’s post hoc comparisons, significantly greater than in UNB plots, where resprouts were absent (Figure 4d; see Table S2 for details).

The Kruskal–Wallis test revealed statistically significant differences among conditions for the percentage of living unburned trees (Figure 4e). The average percentages of living unburned trees in NR and HR (13.33 and 2.86%, respectively) were, for the Dunn’s post hoc comparisons, significantly lower than in UNB and EDG plots (100 and 80.24%, respectively) (Figure 4e; see Table S3 for details).

Likewise, the percentage of living burned trees showed statistically significant differences among conditions, according to the Kruskal–Wallis test (Figure 4f). Dunn’s post hoc comparisons showed statistically significant differences (*p* < 0.05) between UNB (0.00%) and NR (44.58%), UNB and HR (67.62%), and EDG (13.33%) and NR, and EDG and HR (Figure 4f; see Table S3 for details).

The Kruskal–Wallis test revealed statistically significant differences among conditions for the percentage of dead burned trees (Figure 4g). Dunn’s post hoc comparisons showed statistically significant differences (*p* < 0.05) between UNB (0.00%) and EDG (5.71%), and UNB and NR (27.38%) (Figure 4g), while no statistically significant differences were found between the above-mentioned conditions and HR (14.76%) (see Table S3 for details).

The Kruskal–Wallis test indicated statistically significant differences among conditions in terms of delayed mortality (Figure 4h). Dunn’s post hoc comparisons showed statistically significant differences (*p* < 0.05) between NR and UNB plots, NR and EDG plots, and NR and HR plots (Figure 4h). The average percentage of delayed mortality in NR (14.70%) was significantly higher than in EDG (0.71%), HR (0.48%), and UNB (0.00%) (Figure 4h; see Table S3 for details).

No delayed mortality was observed for the tree species *Ilex aquifolium* L., *Quercus ichnusae* Mossa, Bacch. et Brullo, and *Quercus dalechampii* Ten.; however, during the field surveys, it was possible to observe that all these tree species produced resprouts.

### 2.3. Delayed Mortality at the Plant Species Level: Shrub Layer Species

Considering the shrub layer, no delayed mortality was observed for the selected species [*Arbutus unedo* L., *Crataegus monogyna* Jacq., *Cytisus spinosus* (L.) Lam., *Cytisus villosus* Pourr., *Erica arborea* L., *Myrtus communis* L., *Phillyrea latifolia* L., and *Pistacia lentiscus* L.], except for *Erica arborea* (see Figure S1 for details). In particular, for this species, the Kruskal–Wallis test indicated statistically significant differences among conditions in terms of delayed mortality. Dunn’s post hoc comparisons exhibited statistically significant differences (*p* < 0.05) between UNB and NR plots and UNB and HR plots. The average percentage of delayed mortality in UNB (9.55%) was significantly higher than in NR (3.41%) and HR (0.33%).

**Figure 5:**
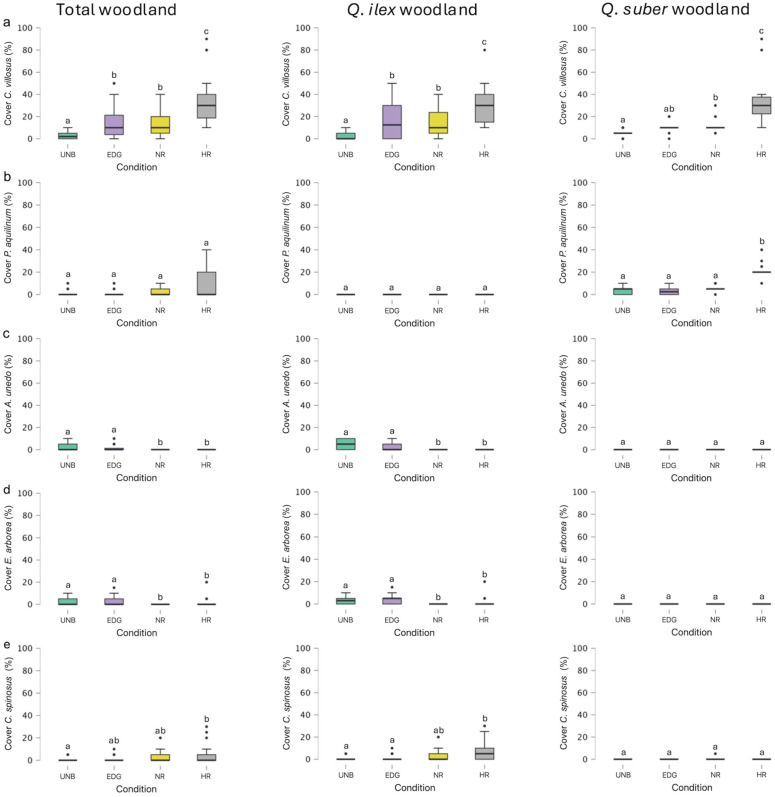
Boxplots of (**a**) *Cytisus villosus*, (**b**) *Pteridium aquilinum*, (**c**) *Arbutus unedo*, (**d**) *Erica arborea*, and (**e**) *Cytisus spinosus* covers considering the total woodland level, the *Q. ilex* level, and the *Q. suber* level in unburned (UNB) plots, edge plots (EDG), no NBR recovery plots (NR), and high NBR recovery plots (HR). The boxes show the interquartile range, the horizontal bars show median values, and the vertical bars show the top and bottom 25% quartiles. Median values with different lowercase letters significantly differ at *p* < 0.05.

The Kruskal–Wallis test revealed statistically significant differences among conditions in terms of the cover percentage of *Cytisus villosus*, both in the total woodland and in the *Q. ilex* and *Q. suber* woodlands (Figure 5a). For the total woodland and the *Q. ilex* woodland, Dunn’s post hoc comparisons showed statistically significant differences (*p* < 0.05) among the different conditions, except for the comparison between EDG and NR. The greatest average cover percentage of *C. villosus* for both total woodlands and *Q. ilex* woodlands was found in HR plots (33.41 and 31.33%, respectively), followed by EDG plots (14.55 and 16.50%, respectively) and NR plots (13.64% and 14.33%, respectively) (Figure 5a). In the *Q. suber* woodlands, statistically significant differences (*p* < 0.05) were observed between the different conditions, except for the comparisons between UNB and EDG and between EDG and NR (Figure 5a). The greatest average cover percentage of *C. villosus* was found in HR plots (37.86%), followed by NR plots (12.14%) and EDG plots (10.36%) (Figure 5a).

Additionally, the Kruskal–Wallis test revealed statistically significant differences among conditions in the cover percentage of *Pteridium aquilinum* (L.) Kuhn, but only in the *Q. suber* woodlands (Figure 5b). Consequently, no statistically significant differences were found (*p* > 0.05) considering the cover percentage of *P. aquilinum* for total woodlands and *Q. ilex* woodlands. Dunn’s post hoc comparisons showed statistically significant differences in the *Q. suber* woodlands (*p* < 0.05) between the conditions UNB and HR, EDG and HR, and NR and HR (Figure 5b). The greatest average cover percentage of *P. aquilinum* was found in HR plots (21.79%), followed by NR plots (5.71%) and UNB plots (3.21%), while the lowest values were observed in EDG (2.86%) (Figure 5b). For all other shrub species considered, no significant differences were found between the different treatments (*p* > 0.05).

Likewise, the cover percentage of *Arbutus unedo*, according to the Kruskal–Wallis test, showed statistically significant differences among conditions but only in the total woodland and in *Q. ilex* woodland. For the total woodland and the *Q. ilex* woodland, Dunn’s post hoc comparisons showed statistically significant differences (*p* < 0.05) between the different conditions, except for the comparison between UNB and EDG and between NR and HR (Figure 5c). The greatest average cover percentage of *A. unedo*, for both total woodlands and *Q. ilex* woodlands, was found in UNB plots (3.18 and 4.67%, respectively), followed by EDG plots (1.59 and 2.33%, respectively). The species *A. unedo* was not present in the NR and HR plots (Figure 5c).

In terms of the cover percentage of *Erica arborea*, the Kruskal–Wallis test indicated significant differences among the conditions but only in the total woodland and the *Q. ilex* woodland. For the total woodland and the *Q. ilex* woodland, the Dunn’s post hoc comparisons showed statistically significant differences (*p* < 0.05) among the different conditions, except for the comparisons between UNB and EDG and between NR and HR (Figure 5d). The greatest average cover percentage of *E. arborea*, for both total woodlands and *Q. ilex* woodlands, was found in EDG plots (3.09 and 4.53%, respectively), followed by UNB plots (1.91 and 2.80%, respectively) and HR plots (0.79 and 1.17%, respectively) (Figure 5d). The species *E. arborea* was not found in the NR plot (Figure 5d).

Lastly, the cover percentage of *Cytisus spinosus*, according to the Kruskal–Wallis test, showed statistically significant differences among conditions but only in the total woodland and *Q. ilex* woodland. Considering the total woodland, the Dunn’s post hoc comparisons showed statistically significant differences (*p* < 0.05) only between the plots UNB and HR (Figure 5e). The greatest average cover percentage of *C. spinosus* in total woodlands was found in HR plots (4.32%), followed by NR plots (2.27%), EDG plots (0.91%), and UNB plots (0.34%). Considering the *Q. ilex* woodland, the Dunn’s post hoc comparisons showed statistically significant differences (*p* < 0.05) between the UNB and HR plots and between EDG and HR (Figure 5e). The greatest average cover percentage of *C. spinosus* in total woodlands was found in HR plots (6.33%), followed by NR plots (3.17%), EDG plots (1.33%), and UNB plots (0.50%).

## 3. Discussion

In this study, based on our previous research [17,62], we examined delayed tree and shrub mortality along the edges of a megafire in the Mediterranean region, focusing on areas lacking spectral recovery, often small and confined to the edges of the burned region.

Fire affects plants, communities, and ecosystems through direct (first-order) and indirect (second-order) effects [3,12,74,75,76]. Plant species may tolerate the first-order fire effects in low- and mixed-severity fires. In this circumstance, they must cope with intricate internal and external processes impacting both ecology and physiology (second-order effects). These mechanisms can increase vulnerability to drought, competition, disease, or insect attacks, potentially leading to delayed mortality [3,12,77]. Although fire intensity is frequently lower along the fire edges [65,66,67], surviving trees may still experience physiologic damage, making them more vulnerable to delayed mortality [3,78,79].

In our study area, the fire was more severe in HR areas, killing off a significant portion of the plants’ above-ground components, with no delayed tree mortality observed. In contrast, the fire was less severe in NR areas, allowing many trees to survive initially, but some showed delayed mortality due to direct and indirect fire effects, explaining the decline in NBR one year post-fire. Both NR and HR plots had similar cover and percentage of living trees, suggesting that delayed mortality can cause significant above-ground phytomass loss, comparable to or exceeding immediate fire effects. This aligns with Reilly et al. [69], who discovered that areas initially categorized as low severity or unburned decreased by approximately 38% and frequently persisted in lesser, more fragmented areas due to delayed mortality. Rossetti et al. [62] suggested that decreased biological competition may explain the absence of delayed tree mortality in HR plots, where damaged trees were still alive throughout field surveys. In fact, fire increases space and resource availability for living trees by reducing tree density and competition from nearby trees, shrubs, and herbs. This might help make up for the immediate effects of the fire and increase the short- and medium-term survival chances of damaged trees [3,12,80,81,82]. Because there were more surviving trees in NR areas immediately after the fire, there was more competition amongst nearby plants than in HR areas, resulting in delayed mortality of the most stressed trees [62].

Among all the parameters analyzed in this study, different aspects emerge when considering the plant community level (woodland) and the plant species level.

The community-level gradients highlight significant differences in delayed mortality and regeneration processes between forest types, confirming that fire responses are not homogeneous. The first PCA gradient highlighted a strong distinction between cork oak and holm oak woodlands, indicating different fire responses linked to their ecological traits and fire resilience. The second component, mainly associated with the distribution of HR and NR plots, emphasized the role of fire severity in post-fire dynamics. HR plots showed stronger associations with resprouting-related variables, such as the cover and height of basal resprouts, particularly in holm oak woodlands. This suggests that high-severity fires have promoted holm oak tree and shrub regeneration, possibly because of their ability to resprout from below-ground plant components, a strategy that helps them recover after disturbance [83]. In contrast, NR plots, particularly in holm oak woodlands, were primarily associated with delayed mortality, reflecting the complex ecological process in which trees and shrubs may appear to survive the fire initially but ultimately succumb to cumulative fire-induced stressors, such as heat damage to tissues, loss of water, and nutritional deficits [3,12,76,84]. These patterns suggest that in low-severity fire areas, delayed mortality may significantly influence community structure, with certain species being more vulnerable over time.

The first PCA component revealed that the cover and height of living trees, both burned and unburned, were primarily associated with cork oak woodlands, which exhibited epicormic resprouting, indicating a more resilient recovery process in these areas. This is because cork oak woodlands have species with greater resistance to fire. The association of *P. aquilinum* with cork oak woodlands suggests that this fern species is ecologically associated with this woodland type, where it might be an important post-fire colonizer that benefits from the disturbance regime [85]. Conversely, in holm oak woodlands, the presence of dead burned trees and shrubs underscores the detrimental effects of fire in this woodland type, even at lower intensities, where delayed mortality is a significant factor. The association of *Q. ilex* and *E. arborea* highlights the role of these species in the post-fire regeneration dynamics of holm oak woodlands.

The delayed mortality pattern observed at the community level is confirmed at the species level for both *Q. ilex* and *Q. suber*. One year following the fire, *Q. suber* presents greater coverage than *Q. ilex* in both NR areas and HR areas. This is likely because of its thick bark, which insulates the tree and enhances its fire resistance [86]. Indeed, bark thickness is a key factor of *Q. suber* post-fire response [87]. Tree vulnerability to fire decreases significantly with increasing bark thickness, with trees having bark over 3–4 cm being well protected from heat damage and unlikely to die or suffer from stem mortality [66]. This ability of *Q. suber* to survive fires confirms the results obtained in previous studies (e.g., [86,88]).

The cover of resprouts was higher in HR areas than in NR areas for both *Q. ilex* and *Q. suber*. Parra and Hinojosa [83] found similar results, with *Q. ilex* showing more shoot growth after high-severity fires compared to areas burned at lower severity or not burned at all. This may be due to reduced competition in HR areas or to the different availability of nutrients in the two post-fire areas. A more severe fire could have released more nutrients into the soil due to the complete combustion of the biomass, favoring a faster initial growth of the resprouts compared to the less affected areas [89,90,91]. However, further analyses are needed to fully understand these dynamics. In addition, the cover of resprouts was greater for *Q. ilex* than for *Q. suber* in both NR and HR areas. This ability allows the species to persist in subsequent generations, so it can be assumed that *Q. ilex* will be able to rebalance its cover with that of *Q. suber*, starting from the regrowth, in the coming years.

The percentage of living unburned plants was low in both the NR and HR areas for both *Q. ilex* and *Q. suber*. In particular, the highest percentage was in NR areas for *Q. suber*. This may be because in these marginal areas, where the fire was less severe, adult individuals of *Q. suber* with thicker bark were not affected by the direct and indirect effects of the fire. Additionally, the percentage of living burned plants was higher for *Q. suber* than for *Q. ilex*, both in NR and HR areas, confirming the greater fire resistance of *Q. suber*.

Delayed mortality was higher in both *Q. ilex* and *Q. suber* in NR areas compared to HR areas. In NR areas, delayed mortality was 25.00% for *Q. ilex* and 14.70% for *Q. suber*, while in HR areas, it was 2.67% for *Q. ilex* and 0.48% for *Q. suber*. These findings align with Curt et al. [86], who reported a percentage of *Q. suber* mortality between 3% and 8% one year after the fire in the Maures massif (France), making this species the most fire-resistant and fire-resilient oak species [86]. In our case, both species follow the overall woodland pattern. This pattern could have the same explanation as previously mentioned: after the fire, there was more competition among nearby plants in NR areas due to a higher density of surviving trees, resulting in delayed mortality of the most stressed trees [62]. Delayed mortality was more expressed in *Q. ilex* than in *Q. suber*. In *Q. suber*, delayed mortality mainly affected individuals whose cork had been harvested, resulting in a lack of fire protection [87].

Species of the shrub layer, particularly *E. arborea*, also exhibited the delayed mortality phenomenon. However, *E. arborea* in the undergrowth usually dies on its own when the woodland canopy closes, making it difficult to assess how much this factor affects the delayed mortality of *E. arborea* in comparison to the impacts of fire. Indeed, delayed mortality is more common in older trees (>100 years) and fire-resistant species, such as *Quercus* spp. [92].

Another result of this study concerns *Cytisus villosus* and *Pteridium aquilinum*. Regarding *C. villosus*, it showed increased cover in high-severity fire areas, both in total woodlands and in *Q. ilex* and *Q. suber* woodlands. The highest cover values were always found in HR areas, confirming the finding reported by Rossetti et al. [17]. This increase may be due to the high post-fire regeneration capacity of the *Cytisus* species [93] and aligns with Xofis et al. [13], who observed that this opportunistic species can become dominant after a fire. *Pteridium aquilinum* showed an increase in the coverage across the total woodlands, with the highest coverage found in HR areas, especially in *Q. suber* woodlands. In fact, *P. aquilinum* has been noted for its tolerance to high-severity fire due to several characteristics [94,95,96,97]. This result can be explained by the fact that *P. aquilinum*, a cosmopolitan species already naturally present in these woodlands, is capable of rapidly recolonizing burned areas. This ability is attributed to its fire-resistant spores, long underground rhizomes that store carbohydrates (which are also immune to fire). Additionally, its survival and spread are favored by specific adaptations, such as strict seasonal periodicity, with a long dormancy period during the dry (burning) season [85,98,99]. Consequently, the environment after the fire is advantageous to its survival and spread [85,100].

## 4. Materials and Methods

### 4.1. Study Area

**Figure 6:**
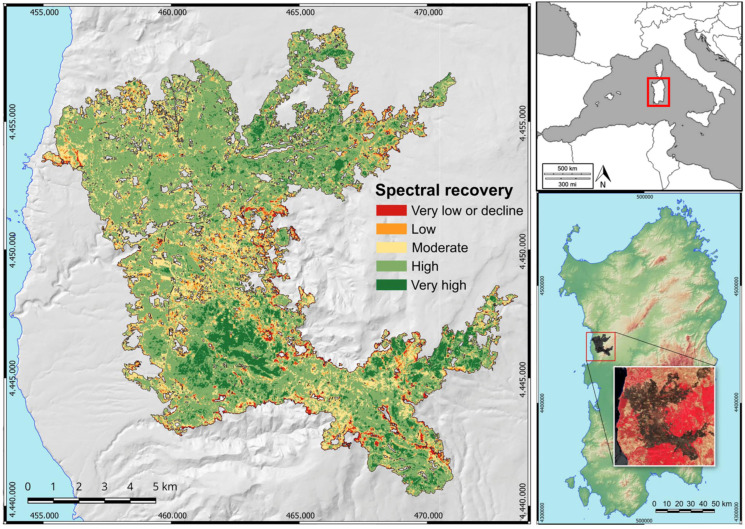
Study area. Map of the spectral recovery levels (based on dNBR) one year after the fire. The areas colored red, i.e., the patches with very low NBR recovery or NBR decline, are the areas of interest in this study. The reference system is WGS84-UTM. In the image at the top right is the position of Sardinia (Italy). While, at the bottom right, the focus map of Sardinia with the location of the study area is visible.

The study area (40°17′/40°6′ N–8°28′/8°42′ E) is in the Montiferru massif (CW Sardinia) (Figure 6). The topography of this massif features a massif derived from a Plio-Pleistocene shield volcano encircled by a vast volcanic plateau. The relief of Montiferru stands out within the Plio–Pleistocene volcanic landscape, which consists of rhyolites and phonolites intersected by trachybasalt dykes, as well as alkaline and hawaiite basalts and lava flows of alkaline and transitional basalts that compose the plateaus of Campeda and Abbasanta [101].

The Montiferru massif is sheer, reaching elevations above 1000 m, with Mt. Urtigu (1050 m a.s.l.) as its highest peak. The area experiences a typical Mediterranean climate, with average annual temperatures ranging from 8.8 °C in January to 24.6 °C in August and an average annual rainfall of 739.3 mm.

According to the Rivas-Martinez classification system [102], the bioclimate is Mediterranean pluviseasonal oceanic, upper thermomediterranean to upper mesomediterranean belts, and upper dry to lower humid. The natural potential vegetation is attributable to broad-leaved woodlands dominated by *Quercus ilex* L. and *Q. suber*, along with mixed forests with deciduous oaks. The landscape is a diverse mosaic of semi-natural vegetation and land-use types, where semi-natural grasslands and fodder crops, linked to livestock farming, cover 49% of the area. Woodlands and Mediterranean shrublands are also widespread, accounting for 20% and 16%, respectively. The remaining 15% includes cultivations, reforestations, urban areas, infrastructure, and other human-altered spaces [103]. In late July 2021, from the 23rd to the 28th, this region experienced a “megafire” (according to Linley et al. [104]) that burned 12,235.5 hectares [17].

### 4.2. Background and Experimental Design: Species Selection and Field Data Collection

Based on the methodology employed in our previous studies [17,62], which utilized the normalized burn ratio (NBR) and the differenced normalized burn ratio (dNBR) indices, we investigated delayed mortality in relation to woodland type and the structural species selected ad hoc for this study. The raster map of spectral recovery, recorded one year following the fire [17], was used to recognize burned areas exhibiting very low NBR recovery or NBR decline. This 10 m resolution raster map was generated from Copernicus Sentinel-2 multispectral data, utilizing the normalized burn ratio (NBR) and the differenced normalized burn ratio (dNBR) as spectral indices. To compute the dNBR, Cloudless Sentinel-2 images [105] acquired on 30 July 2021 and 1 August 2022 were used. To avoid redundancy, we refer to Rossetti et al. [17,62] for a detailed description of the methodology used to generate the spectral recovery map.

Comprehensive vegetation assessments were carried out in the field across woodlands under four distinct conditions, with site selections performed using QGIS software version 3.28: (1) burned woodlands exhibiting low NBR recovery or NBR decline (NR); (2) burned woodlands displaying high NBR recovery (HR); (3) woodlands outside the burned area but very close to the edge of NR areas (EDG); and (4) unburned woodlands (UNB). The categorization of NR, HR, EDG, and UNB areas was established based on the dNBR thresholds (see Table S4 for more details).

A detailed description of the selection of sampling sites is reported in Rossetti et al. [62]. NR woodland areas were identified in sites displaying very low NBR recovery and NBR decline one year following the fire. HR areas were recognized in sites with high and very high spectral recovery one year post-fire, within a 100 m zone around NR areas to reduce spatial distance variations. Both NR (non-recovery) and HR (high-recovery) areas were located within the burned area, mapped using the dNBR index as described in Rossetti et al. [17]. EDG (edge) and UNB (unburned) areas were located outside the burned area. EDG areas were defined as zones apparently unaffected by fire based on dNBR values, located in close proximity to the burned area edge (no farther than 20 m beyond the boundary and within 50 m from NR areas). UNB areas were situated in unburned woodlands, at least 100 m but no more than 200 m from the fire perimeter, again to minimize differences in spatial distance. Both EDG and UNB areas were used as controls within the study.

This configuration enabled us to traverse the gradients at the boundaries of the burned area, from areas with high spectral recovery, through areas with no spectral recovery near the fire edges, to areas just beyond the burned area, and ultimately to areas unaffected by the fire (see Figure S2 for details). Random points were projected with a density of one point per ha and a minimum distance of 50 m between points within the identified areas to establish field sampling areas (see Figure S2 for details).

Surveys were conducted in late spring 2023 to guarantee the maximum vegetative growth. The mapped points were reached in the field using a GPS and Galileo receiver, the Garmin^®^ Montana^®^ 750i (Garmin Ltd., Southampton, UK). Once at the sampling site, a plot of roughly 200 m^2^ was defined, which became the survey area, and for each of these areas an ad hoc field sheet was compiled. The field sheet included, in addition to the code on the station, the date, the detectors, and the woodland type, two different sections dedicated to exploring the post-fire regeneration and the delayed mortality, respectively. Specifically, each section included the following parameters as described below:

1—Post-fire regeneration: the cover of living vegetation, the cover of resprouts only, the mean height of living vegetation, and the mean height of resprouts only were detected for each plot, both for the overall vegetation and for individual species, considering the main tree and shrubby species that determine the physiognomy of the investigated woodlands. In our protocol, the cover of living vegetation provides a direct indicator of the ecosystem’s resistance and resilience after the fire, offering insights into its ability to restore biomass and vegetation cover through epicormic resprouting. Similarly, the cover of resprouts only allows us to understand the contribution of basal vegetative regeneration and seed-based recovery, as opposed to epicormic resprouting, to post-fire recovery. The mean height of living vegetation, in our protocol, acts as an indicator of the structural development of the plant community, helping to evaluate the ability to restore biomass and vegetation cover through epicormic resprouting. Finally, the mean height of resprouts allows us to assess basal vegetative regrowth success and seed-based recovery, as opposed to epicormic resprouting, facilitating comparisons of growth rates among species or areas with varying disturbance intensities.

2—Delayed mortality: the percentage of living plants, the percentage of burnt but still alive plants, the percentage of carbonized dead plants, and the percentage of dead but not carbonized plants were calculated for the forest and shrubby layers separately and for the individual species selected as described above. In our protocol, the percentage of living plants represents the proportion of survived plants that continue to grow, providing a measure of the plant species’ and community’s resilience. The percentage of burned but still alive plants provides a measure of the tolerance of plants to fire and their potential for subsequent regeneration. In contrast, the percentage of carbonized dead plants in our protocol reflects the direct damage caused by the fire, highlighting the severity of the burn. Lastly, the percentage of dead but not carbonized plants allows us to measure delayed mortality, which can occur months or even years after the fire. It helps differentiate the indirect effects of fire (e.g., water stress, competition, infections) from direct heat-related damage. To measure delayed mortality more accurately, it was defined as the percentage of plants that survived the fire or seemed unaffected but died later. The distinction between plants that were instantly killed by the fire and those affected by delayed mortality was based on an assessment of their overall condition. Plants that were fully scorched and bare were regarded as immediately killed, while those without blackened stems and branches, or with minimal fire damage but recently dead leaves, were categorized as undergoing delayed mortality. It is essential to highlight that only the overground parts of the plants were included in these evaluations. As such, the percentages of dead plants reflect top-killing, meaning the fire killed only the above-ground portions.

Considering the practical challenges in the field, each survey was carried out by three surveyors, and the value reported for each parameter was the average of the different evaluations. In total, 176 plots were examined (44 plots per condition) (see Figure S3 for details).

### 4.3. Data Analysis

All cartography computations aimed at selecting sampling sites were performed using QGIS, version 3.28 [106]. To explore relationships among structural plant species, NR and HR conditions, and woodland types, a principal component analysis (PCA) was performed using the Jamovi module Multivariate Exploratory Data Analysis (MEDA), version 2.6.2.0 [107]. To perform the PCA, the 12 parameters collected for the tree and shrub layer, as described above, were used as active variables, while species-specific parameters were used as quantitative supplementary variables. NR and HR conditions, along with woodland types, were included as categorical supplementary variables. Bartlett’s test of sphericity was applied to assess whether the active variables were sufficiently correlated to justify the application of PCA. The Kaiser–Meyer–Olkin (KMO) test was conducted to evaluate the sampling adequacy of the active variables and to exclude those with a KMO value below 0.5. Prior to performing PCA, the variables were normalized by scaling them to unit variance. Varimax rotation was applied to facilitate the interpretation of the principal components.

To examine variations among the conditions (NR, HR, EDG, and UNB), the nonparametric Kruskal–Wallis tests, followed by Dunn’s post hoc comparisons, were used. Prior to conducting these tests, the homogeneity of variance was assessed using Levene’s test. Bonferroni correction was applied to the post hoc comparisons to obtain more conservative *p* values. The analyses were performed in JASP [108].

## 5. Conclusions

This study analyzed the delayed mortality of trees and shrubs along the edges of a Mediterranean megafire, highlighting the influence of fire severity on post-fire resilience. In low-severity (NR) areas, many trees survive at first but suffer a delayed mortality due to the direct and indirect effects of the fire. On the contrary, no delayed tree mortality was seen in HR areas, where the fire was more intense and killed off a large percentage of the above-ground plant components. As expected, cork oak (*Quercus suber*) was more fire-resistant than holm oak (*Q. ilex*) due to its thicker bark and epicormic regrowth capacity. Some shrub species, such as *Erica arborea*, exhibited delayed mortality. Species, such as *Cytisus villosus* and *Pteridium aquilinum*, increased their cover in HR sites; this increase may be due to the high post-fire regeneration and recolonization capacities of these species. These findings highlight the complexity of post-fire dynamics and the importance of considering both species-specific traits and community processes to understand the resilience of Mediterranean ecosystems to fire. The practical implications of these results are relevant for fire management and habitat conservation strategies. Differences in species resistance to fire, such as the greater resistance of *Q. suber* compared to *Q. ilex*, should be considered in forest management practices and fire prevention. However, further studies are necessary, in particular long-term monitoring, to fully understand these complex dynamics and vegetation responses following a wildfire.

## Data Availability

The original contributions presented in this study are included in the article/supplementary material. Further inquiries can be directed to the corresponding author(s).

## Supplementary Materials

The following supporting information can be downloaded at: https://www.mdpi.com/article/10.3390/plants14050730/s1, Table S1: Correlation between the variables and the first dimension (axis 1) and the second dimension (Axis 2); Table S2: Results of the Kruskal–Wallis one-way analysis of variance on ranks test; Multiple comparisons z’ values for the cover of living trees, the cover of resprouted trees, the height of living trees, and the height of resprouted trees analyzed in the study area for the total woodland, the *Quercus ilex* and the *Q. suber* woodlands, for the four plots: unburned (UNB), edge (EDG), no NBR recovery (NR), and high NBR recovery (HR). Statistically significant values (*p* < 0.05) are highlighted in bold; Table S3: Results of the Kruskal–Wallis one-way analysis of variance on ranks test; multiple comparisons z’ values for the living unburned trees, the living burned trees, the dead burned trees, and the delayed mortality analyzed in the study area for the total woodland, the *Quercus ilex* and the *Q. suber* woodlands, for the four plots: unburned (UNB), edge (EDG), no NBR recovery (NR), and high NBR recovery (HR). Statistically significant values (*p* < 0.05) are highlighted in bold; Table S4: Meaning of dNBR values (scaled by 1000) as recovery levels. Recovery level thresholds for low, moderate, high, and very high are based on severity classification proposed by the European Forest Fire Information Service [109]. For the unrecovered/very low and decline levels, thresholds are based on Key and Benson [110]; Figure S1: Boxplots of living unburned, living burned, dead burned, and delayed mortality in unburned (UNB) plots, edge plots (EDG), no NBR recovery plots (NR), and high NBR recovery plots (HR) for the selected shrub species *Crataegus monogyna* Jacq., *Arbutus unedo* L., *Phillyrea latifolia* L., *Cytisus villosus* Pourr., *Erica arborea* L., *Pistacia lentiscus* L., *Myrtus communis* L., and *Cytisus spinosus* (L.) Lam. The boxes show the interquartile range, the horizontal bars show median values, and the vertical bars show the top and bottom 25% quartiles. Median values with different lowercase letters significantly differ at *p* < 0.05; Figure S2: Example of a fire edge showing areas with very low NBR recovery or NBR decline (red areas). Superimposed symbols indicate sampling plots located across the edge; Figure S3: Location of the plots along the edges of the study area (a). All plots are mainly located in the northen (b) and southern (c) part of the burned area in 2021.
