# Supplementary material for: Delayed Vegetation Mortality After Wildfire: Insights from a Mediterranean Ecosystem
Source: Plants (Basel). 2025 Feb 27;14(5):730. doi: 10.3390/plants14050730 (PMC11902081; doi:10.3390/plants14050730)
Supplement: Supplementary file 1 [file plants-14-00730-s001.zip › plants-3487697-supplementary.pdf]

plants

Delayed Vegetation Mortality After Wildfire: Insights from a Mediterranean Ecosystem

Giulia Calderisi [1], Ivo Rossetti [2], Donatella Cogoni [1] and Giuseppe Fenu[1,*]

[1] Department of Life and Environmental Sciences, University of Cagliari, 09123 Cagliari, Italy; giulia.calderisi@unica.it (G.C.); d.cogoni@unica.it (D.C.)
[2] Research Centre of S. Teresa, ENEA (Italian National Agency for New Technologies, Energy and Sustainable Economic Development), 19032 Lerici, Italy; ivo.rossetti@enea.it
* Correspondence: gfenu@unica.it

Table S1. Correlation between the variables and the first dimension (Axis 1) and the second dimension (Axis 2).

Variable	Axis 1		Axis 2	
	Correlation	P value	Correlation	P value
DbShr	0.8721	<0.001		
DbQ.ileTree	0.7886	<0.001		
LbShr	0.7812	<0.001		
HrQ.ile	0.7535	<0.001	0.4291	<0.001
DbE.arb	0.7135	<0.001		
LbE.arb	0.6893	<0.001		
DbTree	0.6693	<0.001		
HrE.arb	0.6314	<0.001	0.2842	<0.001
DMQ.ileTree	0.6077	<0.001	-0.3312	<0.001
DbQ.ileSap	0.5977	<0.001	0.2604	<0.001
DMShr	0.5836	<0.001	-0.3533	<0.001
CrE.arb	0.545	<0.001		
DMQ.ileSap	0.5154	<0.001	-0.2445	<0.001
LbQ.ileTree	0.4711	<0.001	0.6423	<0.001
CrQ.ile	0.4651	<0.001	0.6659	<0.001
LbQ.ileSap	0.3867	<0.001	0.2922	<0.001
HLC.spi	0.3845	<0.001		
DMTree	0.3569	<0.001	-0.4398	<0.001
HrA.une	0.3464	<0.001	0.2356	<0.001
DbA.une	0.3323	<0.001		
Cr	0.3235	<0.001	0.7994	<0.001
CLC.spi	0.2967	<0.001	-0.2179	<0.001
HLQ.ile	0.2757	<0.001		
CrA.une	0.2575	<0.001		
LbA.une	0.2551	<0.001	0.287	<0.001
LC.spi	0.2527	<0.001		
DMQ.ich	-0.2509	<0.001		
LuQ.ich	-0.2777	<0.001	-0.3054	<0.001
LbQ.ich	-0.3111	<0.001		
LuTree	-0.3392	<0.001	-0.2981	<0.001
HLC.vil	-0.3791	<0.001		
CrQ.ich	-0.3921	<0.001		
DbQ.sub	-0.4019	<0.001		
LuQ.sub	-0.4166	<0.001	-0.2911	<0.001

 https://doi.org/10.3390/plants14050730

CLQ.ich	-0.4265	<0.001	-0.2864	<0.001
HrQ.ich	-0.4727	<0.001		
DbQ.ich	-0.474	<0.001		
HLQ.ich	-0.4778	<0.001		
LC.vil	-0.488	<0.001		
DMQ.sub	-0.4943	<0.001		
CrQ.sub	-0.5656	<0.001		
LbTree	-0.6076	<0.001	0.5854	<0.001
CP.aqu	-0.725	<0.001		
CL	-0.761	<0.001		
HrQ.sub	-0.7753	<0.001		
HL	-0.7908	<0.001		
LbQ.sub	-0.8186	<0.001		
CLQ.sub	-0.8258	<0.001		
HLQ.sub	-0.8521	<0.001		
LP.aqu	-0.8686	<0.001		
HLP.aqu	-0.9016	<0.001		
LuShr	-0.9301	<0.001		
Hr			0.829	<0.001
HLE.arb			0.4919	<0.001
CsE.arb			0.4129	<0.001
CsQ.ile			0.291	<0.001
DME.arb			-0.2662	<0.001

CL = Cover of living plants, HL = Height of living plants, Cr = Cover of resprouts, Hr = Height of resprouts, Lb = Living burned plants, Lu = Living unburned plants, L = living plants (for *Pteridium aquilinum*, *Cytisus villosus* and *Cytisus spinosus* only), Db = Dead burned plants, DM = Delayed mortality, Tree = Arboreal plants, Shr = Shrubs, Sap = Sapling, Q.ile = *Quercus ilex*, Q.sub = *Q. suber*, Q.ich = *Q. ichnusae*, E.arb = *Erica arborea*, A.une = *Arbutus unedo*, P.aqu = *Pteridium aquilinum*, C.vil = *Cytisus villosus*, C.spi = *Cytisus spinosus*.

Table S2. Results of the Kruskal–Wallis One-Way Analysis of Variance on Ranks test; Multiple Comparisons z' values for the cover of living trees, the cover of resprouts trees, the height of living trees, and the height of resprouts trees analyzed in the study area for the total woodland, the *Quercus ilex* and the *Q. suber* woodlands, for the four plots: Unburned (UNB), Edge (EDG), no NBR recovery (NR), and high NBR recovery (HR). Statistically significant values ($p < 0.05$) are highlighted in bold.

		Cover of living trees					Cover of resprouts trees			
		UNB	EDG	NR	HR		UNB	EDG	NR	HR
**Total woodland**	UNB		**3.921**	**8.430**	**7.381**	UNB		**-3.579**	**-7.673**	**-8.833**
	EDG			**4.509**	**3.460**	EDG			**-4.094**	**-5.254**
	NR				-1.049	NR				-1.160
	HR					HR				
***Q. ilex* woodland**	UNB		**3.271**	**8.618**	**6.360**	UNB		**-2.894**	**-6.980**	**-7.595**
	EDG			**5.346**	**3.089**	EDG			**-4.086**	**-4.700**
	NR				-2.258	NR				-0.615
	HR					HR				
***Q. suber* woodland**	UNB		2.168	**3.754**	**4.484**	UNB		-2.025	**-3.377**	**-4.811**
	EDG			1.586	2.316	EDG			-1.352	**-2.786**
	NR				0.731	NR				-1.434
	HR					HR				

		Height of living trees					Height of resprouts trees			
		UNB	EDG	NR	HR		UNB	EDG	NR	HR
**Total woodland**	UNB		1.996	**7.395**	**7.823**	UNB		**-3.959**	**-7.410**	**-8.841**
	EDG			**5.400**	**5.828**	EDG			**-3.452**	**-4.882**
	NR				0.428	NR				-1.430
	HR					HR				
***Q. ilex* woodland**	UNB		1.221	**-6.876**	**-6.839**	UNB		**-3.205**	**-6.577**	**-7.782**
	EDG			**-5.655**	**-5.618**	EDG			**-3.372**	**-4.578**
	NR				-0.037	NR				-1.206
	HR					HR				
***Q. suber* woodland**	UNB		2.450	**2.847**	**3.804**	UNB		-2.286	**-3.566**	**-4.290**
	EDG			0.397	1.354	EDG			-1.280	-2.005
	NR				0.957	NR				-0.724
	HR					HR				

Table S3. Results of the Kruskal–Wallis One-Way Analysis of Variance on Ranks test; Multiple Comparisons z' values for the living unburned trees, the living burned trees, the dead burned trees, and the delayed mortality analyzed in the study area for the total woodland, the *Quercus ilex* and the *Q. suber* woodlands, for the four plots: Unburned (UNB), Edge (EDG), no NBR recovery (NR), and high NBR recovery (HR). Statistically significant values ($p < 0.05$) are highlighted in bold.

		**Living unburned trees**					**Living burned trees**			
		UNB	EDG	NR	HR		UNB	EDG	NR	HR
**Total woodland**	UNB		**3.220**	**9.469**	**10.338**	UNB		**-2.874**	**-7.776**	**-8.975**
	EDG			**6.249**	**7.119**	EDG			**-4.902**	**-6.101**
	NR				0.870	NR				-1.199
	HR					HR				
***Q. ilex* woodland**	UNB		2.430	**8.218**	**8.344**	UNB		-1.791	**-6.183**	**-7.577**
	EDG			**5.788**	**5.914**	EDG			**-4.391**	**-5.786**
	NR				0.126	NR				-1.394
	HR					HR				
***Q. suber* woodland**	UNB		2.119	**5.000**	**6.214**	UNB		-1.960	**-4.650**	**-5.363**
	EDG			**2.881**	**4.095**	EDG			**-2.690**	**-3.403**
	NR				1.214	NR				-0.713
	HR					HR				

		**Dead burned trees**					**Delayed mortality**			
		UNB	EDG	NR	HR		UNB	EDG	NR	HR
**Total woodland**	UNB		**-3.657**	**-7.895**	**-7.465**	UNB		-2.060	**-9.117**	-1.159
	EDG			**-4.238**	**-3.808**	EDG			**-7.057**	0.901
	NR				0.430	NR				**7.958**
	HR					HR				
***Q. ilex* woodland**	UNB		**-2.658**	**-6.676**	**-7.322**	UNB		-1.776	**-7.470**	-0.915
	EDG			**-4.018**	**-4.664**	EDG			**-5.694**	0.861
	NR				-0.646	NR				**6.555**
	HR					HR				
***Q. suber* woodland**	UNB		**-2.875**	**-4.412**	-2.162	UNB		-0.916	**-5.530**	-0.601
	EDG			-1.537	0.712	EDG			**-4.614**	0.315
	NR				2.250	NR				**4.929**
	HR					HR				

Table S4. Meaning of dNBR values (scaled by 1000) as recovery levels. Recovery level thresholds for Low, Moderate, High and Very high are based on severity classification proposed by the European Forest Fire Information Service [1]. For the Unrecovered/Very Low and Decline levels, thresholds are based on Key and Benson [2].

dNBR values	Recovery level	Meaning
dNBR ≥ +100	Decline	Phytomass loss
+99 ≥ dNBR ≥ −100	Unrecovered/Very low	Little or no changes
−101 ≥ dNBR ≥ −255	Low	Vegetation regrowth
−256 ≥ dNBR ≥ −419	Moderate	
−420 ≥ dNBR ≥ −660	High	
dNBR < −660	Very high	

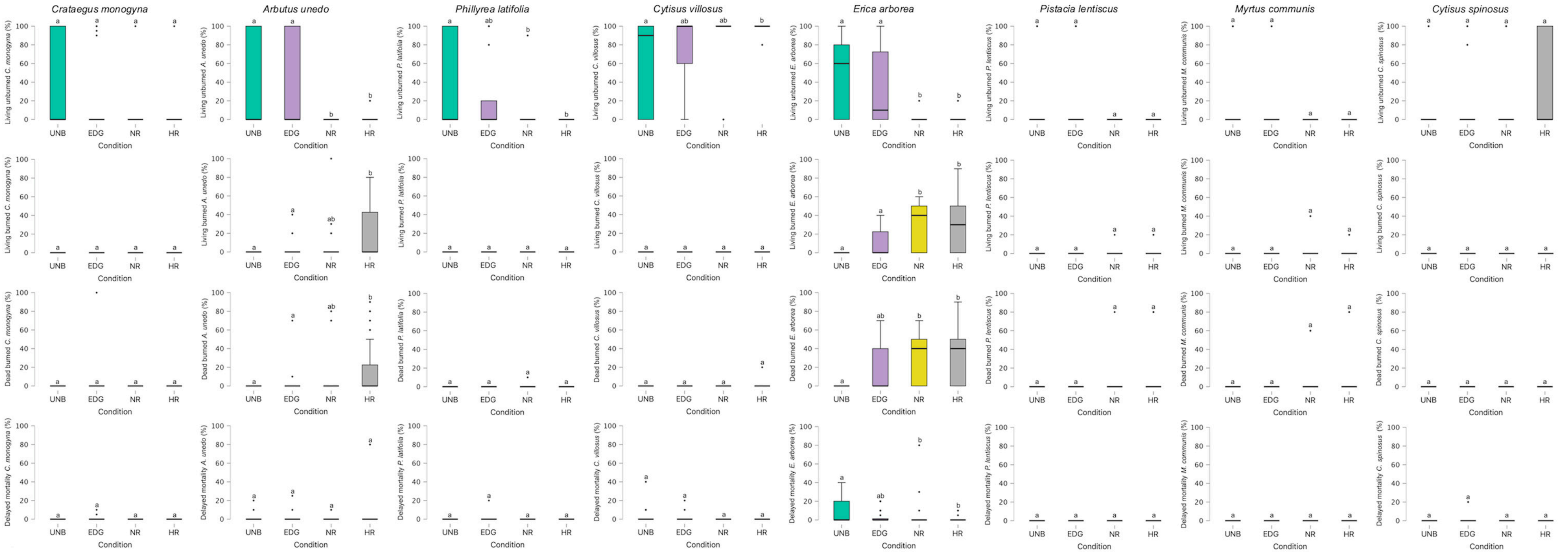

Figure S1. Boxplots of living unburned, living burned, dead burned, and delayed mortality, in unburned (UNB) plots, edge plots (EDG), no NBR recovery plots (NR), and high NBR recovery plots (HR) for the selected shrub species *Crataegus monogyna* Jacq., *Arbutus unedo* L., *Phillyrea latifolia* L., *Cytisus villosus* Pourr., *Erica arborea* L., *Pistacia lentiscus* L., *Myrtus communis* L., and *Cytisus spinosus* (L.) Lam. The boxes show the interquartile range, the horizontal bars show median values, and the vertical bars show the top and bottom 25% quartiles. Median values with different lowercase letters significantly differ at $p < 0.05$.

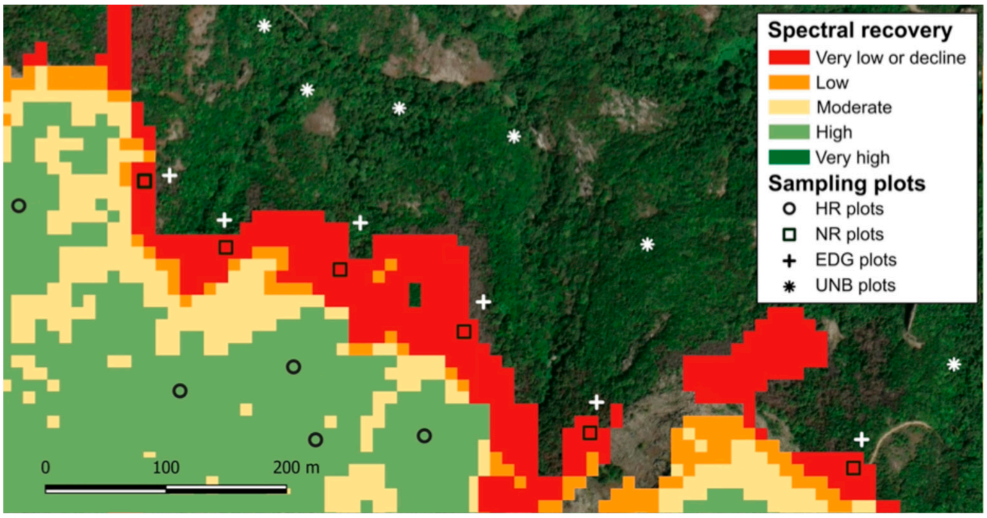

Figure S2. Example of a fire edge showing areas with very low NBR recovery or NBR decline (red areas). Superimposed symbols indicate sampling plots located across the edge.

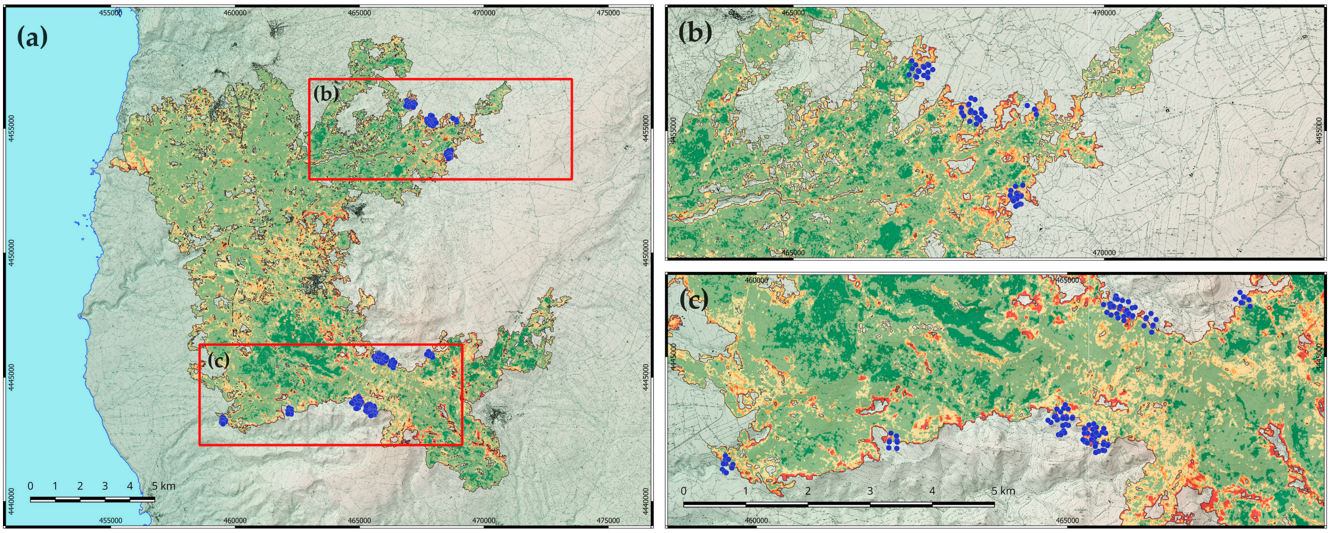

Figure S3. Location of the plots along the edges of the study area (a). All plots are mainly located in the Northen (b) and Southern (c) part of the burned area in 2021.

References

1. European Forest Fire Information System (EFFIS). Available online: https://effis.jrc.ec.europa.eu/about-effis/technical-background/rapid-damage-assessment (accessed on 20 August 2022).
2. Key, C.H.; Benson, N.C. Landscape Assessment (LA) Sampling and Analysis Methods. In FIREMON: Fire Effects Monitoring and Inventory System; USDA Forest Service, Rocky Mountain Research Station: Ogden, UT, USA, 2006.
